# Are Hepatic Adenomas Premalignant?

**DOI:** 10.1155/1996/59629

**Published:** 1996

**Authors:** David M. Nagorney

**Affiliations:** Department of Surgery Mayo Clinic 200 First Street Southwest Rochester Minnesota 55905 USA

## Abstract

*Objective*: To investigate clinical experience with the apparent malignant transformation of benign liver cell adenomas.

*Design*: Retrospective review of personal experience and literature.

*Setting*: University hospital and affiliated community hospitals.

*Patients*: All patients diagnosed with liver cell adenomas over a 30-year period.

*Interventions*: Liver resection and/or tumor biopsy.

Main Outcome Measures: Gender, age, drug associations, alpha-fetoprotein levels, response to treatment, and survival.

*Results*: Thirteen patients from personal experience and 26 patients from the reports of others had liver cell adenomas that were not resected. Five of these patients subsequently developed hepatocellular carcinoma.

*Conclusions*: Malignant transformation of a liver cell adenoma is a rare phenomenon, but it does occur. Alphafetoprotein levels may be more helpful in diagnosis than expected from previous reports. Solitary benign adenomas should be resected whenever possible. Patients with diffuse multiple tumors should be observed closely over a long period.


*HPB Surgery*, 1996, Vol.10, pp.59–63
Reprints available directly from the publisher
Photocopying permitted by license only

© 1996 OPA (Overseas Publishers Association)
Amsterdam B.V. Published in The Netherlands
by Harwood Academic PublishersGmbH
Printed in Malaysia

**HPB INTERNATIONAL**

**EDITORIAL & ABSTRACTING SERVICE**

JOHN TERBLANCHE, EDITOR
Department of Surgery,
Medical School · Observatory 7925
Cape Town · South Africa
Telephone: 27-21-406-6204
Telefax 27-21-448-6461
E-mail: JTER BLAN@UCT GSHI.UCT.AC.2A

### ARE HEPATIC ADENOMAS PREMALIGNANT?

### ABSTRACT

*Foster, J.H. and Berman, M.M. (1994) The malignant transformation of liver cell adenomas. Arch Surg.,* ***129:*** *717–717.*

***Objective*: To investigate clinical experience with the apparent malignant transformation of benign liver cell adenomas.**
***Design*: Retrospective review of personal experience and literature.**
***Setting*: University hospital and affiliated community hospitals.**
***Patients*: All patients diagnosed with liver cell adenomas over a 30-year period.**
***Interventions*: Liver resection and/or tumor biopsy.**
**Main Outcome Measures: Gender, age, drug associations, alpha-fetoprotein levels, response to treatment, and survival.**
***Results*: Thirteen patients from personal experience and 26 patients from the reports of others had liver cell adenomas that were not resected. Five of these patients subsequently developed hepatocellular carcinoma.**
***Conclusions*: Malignant transformation of a liver cell adenoma is a rare phenomenon, but it does occur. Alphafetoprotein levels may be more helpful in diagnosis than expected from previous reports. Solitary benign adenomas should be resected whenever possible. Patients with diffuse multiple tumors should be observed closely over a long period. *Arch Surg*. 1994; 129: 712–717**

KEY WORDS: Hepatic adenoma hepatocellular carcinoma

### PAPER DISCUSSION

Doctors James Foster and Martin Berman, both respected and recognized authorities of hepatic tumors in their respective fields, have examined the reported clinical evidence for the malignant transformation of liver cell adenomas (LCA). In brief, they culled 39 patients with LCAs that were not resected or were incompletely resected from their own experience or from other reports. Five patients developed hepatocellular carcinoma after the diagnosis of LCA. These findings, at face value, suggest that nearly 13% of patients with unresected LCA risk malignant degeneration. However, Foster and Berman interestingly conclude that malignant transformation is "a rare phenomenon". Although their conclusion may seem incongruous with the absolute value of their finding, their analysis is convincingly expounded.

This original article examined several reputed associations between LCA and malignant transformation: number of LCAs, contraceptive steroid use, alpha fetoprotein levels, and the diagnostic interval between confirmation of benign and malignant hepatocellular histology. None of these factors were predicative of malignant transformation. Cessation of steroid use did not abort the risk of malignant tranformation. Alphafetoprotein level was not a consistent marker of malignancy. Patients with multiple LCAs were too few to assess and diagnostic intervals between the LCA and HCC were marginal (3.5 years or less) in two patients. Despite illusions to the reputed frequency of the malignant potential for LCAs, only five cases were substantive enough to even support the premise. Furthermore, Foster and Berman emphasized that the natural history of LCA is unknown. Of the 22 patients with incompletely or unresected LCAs proven histologically who had reported follow-up, LCAs decreased in size in nearly 2/3 while the remainder were unchanged. The factors relevant to the size decrease of LCAs were indeterminant. Neither rupture nor cancer were recognized and few patients developed any symptoms. The natural history of LCAs in this very selected review was remarkably benign. Clearly, the coincidence of LCA and HCC is rare.

Have the authors provided us with enough evidence to support malignant transformation of this specific hepatic pathology? What basic objective evidence is required for malignant transformation of a benign denoma? First, histologic confirmation (not cytologic) of both benign and malignant liver cell pathology must be established according to accepted pathologic standards. Second, a diagnostic interval of such duration must exist to temporally permit the cellular and gross neoplastic transformation. Finally, imaging data should confirm a single site of hepatic origin. Clearly, histologic evidence is essential and independent pathologic coraboration is optimal. Cytologic interpretation alone is currently an unacceptablestandard for LCA and cannot be included. A reasonable period for malignant transformation remains speculative. Extrapolation from the adenoma-carcinoma sequence of gut malignancies (in which the epthelial turnover rate far exceeds that of liver cells) suggests that at least three to five years minimum is a reasonable duration, though longer periods would even be more convincing. Thirdly, imaging of malignant tumors much arise within the same focus of the liver. Simple convergenceof abutting tumors must be excluded.

This study of Foster and Berman does have several limitations however. First, the total number of patients studied was small (39 patients). Although the expected frequency of malignant transformation should be proportionally expressed in this small population, the chances of observing this event are small. Thus, the sample size has a real chance of a type II statistical error. Second, the duration and type of follow-up after diagnosis of LCA was uncontrolled. Although 9 of 12 patients of Foster and Berman's personal series had follow-up for more than 10 years, the duration of follow-up of the 26 patients collected from the literature is unstated, and is neither as long or as complete as Foster and Berman's personal series. Given the timeframe required to convincingly confirm the adenoma-carcinoma transformation in the colon, the timeframe herein may simply be inadequate to observe the transformation of LCA to HCC. Finally, few if any reported biopsies of observed LCAs were reported. The natural history of small HCC itself is only beginning to be clarified and our knowledge of LCAs is even more primitive. Could some malignant transformations have been missed? Did transformation occur in some patients without clinical recognition? Do HCCs arsing from LCAs have the same prognosis as HCCs arising in cirrhotic liver, non-cirrhotic livers, or do they mimic less agressive HCCs such as fibrolamellar variants? Was the risk of malignancy reduced in some patients because of partial resection?

Rightfully, Foster and Berman relate their findings to the clinical management of patients with asymptomatic LCAs. Albeit rare, they believe that malignant transformation does occur. Long-term follow-up is the minimum responsible management course for most patients. Neither levels of tumor markers nor discontinuation of sex steroids aborts the risk of malignant transformation. Importantly, because operative risk for hepatic resections continues to decline, and, infact, because the mortality rate for resection for benign disease is less than the risk of malignant transformation resection has been advocated for most LCAs, Moreover, given the age of most patients with LCAs, both duration and intensity of follow-up will be costly. Although a cost benefit analysis was not addressed by Foster and Berman, an argument could be made that immediate operation versus the continual escalating cost of repeated liver imaging, serial tumor markers, physical examinations, and health care expense for any patients developing HCC over the course of at least several decades would be more cost effective.

In conclusion, the article is humbling. In their overtly honest and unpretentious evaluation of the meager clinical data available regarding the malignant transformation of LCAs, Foster and Berman clearly confront us with our limited knowledge of LCAs. Despite the paucity of data, they have proposed tentative management guidelines which are reasonable with the proviso that the benefit-risk ratio of operation to observation is overwhelmingly patient favorable. Perhaps our response in appreciation to these authors should be the establishment of a world-wide registry of patients with histologically-proven LCAs to determine their course; only collectively will we answer the questions which they so humbly pose.

David M Nagorney
Department of Surgery
Mayo Clinic
200 First Street Southwest
Rochester
Minnesota 55905
United States of America

### TYPE IVA CHOLEDOCHAL CYST: IS HEPATIC RESECTION NECESSARY?

### ABSTRACT

*Chijiiwa, K., Komura, M. and Kameoka, N. (1994) Postoperative followup of patients with type IVA choledochal cysts after excision of extrahepatic cyst. Journal of The American College of Surgeons; 179: 641–645.*

**_Background:_ This study concerns patients who have choledochal cyst with intrahepatic and extrahepatic involvement (type IVA cyst). The extent of excision and the necessity of hepatectomy, including the intrahepatic cyst in these patients have not been clarified.**
**_Study design:_ We have performed excision of the extrahepatic cyst with hepaticojejunostomy upon 13 patients with type IVA cyst during a 16 year period. The present study was done to examine the size of the anastomotic opening by direct cholangiography two weeks postoperatively. The long-term results were assessed to find the appropriate operative management for patients with type IVA cysts.**
**_Results:_ Intrahepatic cysts were present in both hepatic lobes in 11 patients (85 percent). None of the patients had carcinoma after excision of extrahepatic cyst during the follow-up period, which ranged from two months to 16 years. Postoperative late complications occurred in three patients (23 percent), hepatolithiasis in two and cholangitis in one. The anastomotic opening of hepaticojejunostomy was 13.3±4.5 mm in diameter two weeks postoperatively, which was not significantly different when compared with that in ten patients without late complications (13.4±4.9 mm). The late complications were successfully treated with either antibiotics or percutaneous transhepatic cholangioscopy, and none required a reoperation.**
**_Conclusions:_ The results suggest that additional hepatectomy is not required because carcinoma has rarely occurred from the intrahepatic cyst. Excision of an extrahepatic cyst with a wide hepaticojejunostomy is an acceptable operative management for patients with type IVA cysts. _J. Am. Coll. Surg._, 1994, 179: 641–645.**

KEY WORDS: Choledochal cyst hepatic resection.

